# Protective effect of propofol on hydrogen peroxide-induced human esophageal carcinoma via blocking the Wnt/β-catenin signaling pathway

**DOI:** 10.22038/ijbms.2018.29141.7039

**Published:** 2018-12

**Authors:** Jian-Jun Xue, Ling-Yun Zhang, Huai-Jing Hou, Yan Li, Wan-Sheng Liang, Ke-Hu Yang

**Affiliations:** 1 Evidence Based Medicine Centre, School of Basic Medical Sciences, Lanzhou University, Lanzhou 730000, China; 2 Key Laboratory of Evidence Based Medicine and Knowledge Translation of Gansu Province, Lanzhou 730000, China; 3 Gansu Provincial Hospital of TCM, Lanzhou 730050, China

**Keywords:** Esophageal neoplasms, Hydrogen peroxide, Propofol, Wnt signaling pathway β-catenin

## Abstract

**Objective(s):**

To analyze the potential influences of propofol on the oxidative stress of H_2_O_2_-induced human esophageal squamous cell carcinoma (ESCC) Eca109 cell through mediating the Wnt/β-catenin signaling pathway.

**Materials and Methods:**

Eca109 cells were classified into 5 groups: Control group, H_2_O_2_ group, Propofol + H_2_O_2_ group, Dkk1 (Dickkopf-1, Wnt/β-catenin pathway antagonist) + H_2_O_2_ group, and Propofol + LiCl (Lithium chloride, Wnt/β-catenin pathway agonist) + H_2_O_2_ group. Western blotting was performed to determine the protein expressions, flow cytometry to measure the content of ROS, immunofluorescence staining to detect the oxidative DNA damage, as well as MTT, AnnexinV-FITC/PI, Wound-healing, and Transwell assays to test the biological characteristics of Eca109 cells.

**Results:**

H_2_O_2_ resulted in the increased nuclear and cytoplasmatic expression of β-catenin, reduced p-GSK3β expression, up-regulated ROS content, and induced oxidative DNA damage in Eca109 cells. Moreover, Eca109 cells treated with H_2_O_2_ alone had enhanced cell proliferation and metastasis but decreased cell apoptosis, as compared with those without any treatment; meanwhile, the declined Cyt C, Bax, and cleaved caspase-3, as well as the elevated Bcl-2 were also observed in Eca109 cells in the H_2_O_2_ group, which were reversed by Propofol or Dkk1. Moreover, Propofol could inhibit the effect of LiCl on activating the Wnt/β-catenin signaling pathway in H_2_O_2_-induced Eca109 cells.

**Conclusion:**

Propofol elicits protective effects to inhibit H_2_O_2_-induced proliferation and metastasis and promote apoptosis of Eca109 cells via blocking the Wnt/β-catenin pathway, offering a possible therapeutic modality for ESCC.

## Introduction

Esophageal cancer (EC), arising from the esophagus, ranks as the sixth most fatal cancer around the world, with the rapidly increasing incidence year by year (1). Pathologically, EC can be divided into esophageal adenocarcinoma (EAC) and esophageal squamous cell carcinoma (ESCC), with the latter being the dominant one mainly found in Asian and African regions and the former usually occurring in western countries (2, 3). Although significant progress has been made in EC treatment, the 5-year overall survival rate of ESCC is only about 14% (4). Numerous studies have reported that the accumulation of DNA damage under the condition of long-term oxidative stress would facilitate the aging process and lead to the occurrence of malignancies and degenerative diseases (5, 6). As reported, hydrogen peroxide (H_2_O_2_), as an exogenous active oxygen, can diffuse across membranes to form highly reactive radicals with iron ions in cells through Fenton’s reaction, which is often used to construct various oxidative damage models owing to its stability and easy availability (7, 8). Additionally, the degree of oxidative stress was suggested to affect the occurrence and development of ESCC (9, 10). Hence, it is of great importance and urgency to explore the promising therapeutic target to inhibit the oxidative stress in ESCC.

Propofol (2,6-diisopropylphenol) is commonly accepted as an immediately effective intravenous anesthetic agent in clinical use initially introduced to China in the late 1980s, which can induce and maintain anesthesia in a fast and stable manner, as well as rapid recovery from anesthesia (11). In recent years, propofol has been proven to have various non-anesthetic effects in addition to its multiple advantages as an anesthetic drug (12). For example, propofol can also exert the potential anti-tumor characteristics to affect the proliferation and metastasis of human cancer cells (13, 14). It also has been suggested to have immunomodulatory, antioxidant, analgesic, antiemetic, and neuroprotective properties through its clinical practice(12), highlighting a better option during cancer surgeries when compared to other anesthetic agents (13). Mounting studies have demonstrated the great influence of propofol on cancer development through mediating signaling pathways. For instance, propofol could restrict the proliferation and invasion of HepG2 cells by blocking the Wnt/β-catenin pathway, but facilitate the cell apoptosis, as indicated by Ou *et al *(15). To our knowledge, Wnt signaling is important during organ development and tissue homeostasis involved in the pathogenesis of several diseases, including cancers (16, 17), which can be broadly diversified into canonical or noncanonical pathways (18). The canonical pathway could regulate the stability of transcription co-activator β-catenin, thus involving in human malignancies (19, 20). An increasing amount of evidence demonstrated Wnt/β-catenin was associated with cancer cell proliferation, migration, invasion, and resistance to drugs, suggesting its potential role as a novel cancer therapeutic target (21, 22). Worth mentioning, there was reported linkage between the Wnt/β-catenin pathway and the carcinogenesis of ESCC (23, 24), which was also related to DNA damage response and oxidative stress (25). Nevertheless, whether propofol can regulate the occurrence and progression of ESCC by mediating the Wnt/β-catenin pathway remains unclear. 

As such, we induced the human ESCC cell line Eca109 by H_2_O_2_ and then observed the pre-treatment effect of propofol, dickkopf-1 (Dkk1, wnt/β-catenin pathway antagonist), and lithium chloride (LiCl, Wnt/β-catenin pathway agonist) on the oxidative DNA damage and other biological characteristics of Eca109 cells. 

## Materials and Methods

***Treatment and grouping of Eca109 cells***

Human ESCC cell line Eca109 (American Type Culture Collection, ATCC, Manassas, VA, USA) were cultured in the RPMI-1640 medium (BioWhittaker and Cambrex, Verviers, Belgium) containing 10% inactivated fetal bovine serum (FBS, Gibco BRL, Grand Island, NY, USA), penicillin (100 units/ml, Sigma, St-Louis, MO, USA), and streptomycin (100 mg/ml, Sigma, St-Louis, MO, USA), and incubated in 5% CO_2 _at 37 **°**C. Eca109 cells were classified into Control group (cells without any treatment), H_2_O_2_ group (cells treated by 10 μM H_2_O_2_ for 3 hr), Propofol + H_2_O_2_ group (cells treated with 5 μg/l propofol for 30 min before 3 hr of treatment with 10 μM H_2_O_2_), Dkk1 + H_2_O_2_ group (cells treated with 20 mmol/l Dkk1 for 30 min before 3 hr of treatment with 10 μM H_2_O_2_), and Propofol + LiCl + H_2_O_2_ group (cells treated with 5 μg/l propofol and 20 mmol/l LiCl for 30 min before 3 hr of treatment with 10 μM H_2_O_2_). 

***Western blotting***

A commercial protein isolation kit (TransGenBiotec, Beijing, China) was used to prepare the protein form cytoplasmic and nuclear fractions, and the concentration was determined according to the bicinchoninic acid (BCA) Kit (Thermo Scientific, IL, USA). The proteins were heated with the addition of the loading buffer for 10 min at 95 **°**C. Electrophoresis was applied for the separation of proteins (50 μg/well) in sodium dodecyl sulfate-polyacrylamide gel electrophoresis (SDS-PAGE) gels (Bio-Rad Laboratories, Hercules, California, USA). The resolved proteins were transferred to polyvinylidene difluoride (PVDF) membrane at 120 V for 3 hr, which was placed in 5% bovine serum albumin (BSA) for 1 hr at room temperature, followed by the addition of primary antibodies (Abcam, Cambridge, CA, USA) for overnight incubation at 4 ^°^C, including anti-β-Catenin (ab16051, 0.25 µg/ml dilution), anti-phosphor-GSK3β (ab75745, 1/500 dilution), anti-GSK3β (ab32391, 1/10000 dilution), anti-Cytochrome C (ab13575, 1 µg/ml dilution), anti-Bax (ab32503, 1/2000 dilution), anti-active Caspase-3 (ab2302, 1 µg/ml dilution), anti-Bcl-2 (ab32124, 1/1000 dilution), anti-lamin B1 (ab16048, 1/1000 dilution), and anti-β-actin (ab8226, 1 µg/ml dilution). After the membrane was washed with Tris-buffered saline with Tween 20 (TBST, 3 times/5 min), the corresponding secondary antibodies (Abcam, Cambridge, CA, USA) were added for incubation for 1 hr, followed by another rinse with TBST (3 times/5 min). The development was based on the chemiluminescent (CL) detection. The β-actin and lamin B1 served as cytoplasmic and nuclear protein loading controls, respectively. The experiment was repeated three times to validate the results.

***Measurement of ROS content with flow cytometry***

When cell confluence reached about 80%, cells were digested using 0.2% collagenase and separately inoculated into the 60 mm^2^ culture dishes. When the cell confluence was about 70–80%, they were washed with PBS twice, digested by Trypsin for 2 min, mixed with an appropriate volume of culture medium, and transferred to corresponding Eppendorf (EP) tubes for centrifugation (5 min at 800 g). Then, the supernatant was removed followed by the addition of ROS detection solution (500 μl). At last, cells were re-suspended and left for 30 min of reaction at 37 **°**C in a dark environment, followed by detection in a flowcytometer (Beckman-Coulter, CA, USA). The experiment was done independently three times to test reproducibility.

***Immunofluorescence assay***

Eca109 cells were incubated in the medium containing 10% normal goat serum at room temperature for 20 min, which were then incubated with anti-8-oxoguanineat 4 °C overnight and the secondary antibody conjugated with tetramethylrhodamine isothiocyanate (TRITC) for 45 min at room temperature. After that, slides were mounted for analysis by Vectashield with 4’-6-diamidino-2-phenylindole (DAPI) (Invitrogen, Molecular Probes, Eugene, Oregon, USA) and placed under a fluorescence microscope for observation and photo-taking. The image analysis was performed by using Image J software (National Institutes of Health, USA), and at least 1000 cells were measured each time. For better visualization, the red fluorescence was converted into pseudo green. As regards fluorescein-labeled avidin (FITC-avidin), the 8-Oxoguanine antibody was replaced by FITC-conjugated avidin (1:200, Santa Cruz), and cells were incubated in the dark environment at 37 °C for another 1 hr. The experiment was repeated three times.

***MTT assay***

Eca109 cells at the logarithmic growth phase were collected and inoculated into a 96-well plate (5×10^3^ cells/well), which were cultured at 37 °C with 5% CO_2_ for various time periods (12 hr, 24 hr, 48 hr, and 72 hr). Next, the culture plate was taken out from the incubator followed by the addition of 3-(4,5 Dimethylthiazol -2-1)-2-5-diphenyl tetrazolium bromide (MTT, 50 μl) for another incubation (4 hr). After that, the cells were centrifuged for 10 min at the rate of 2000 rpm, and dimethyl sulfoxide (DMSO, 200 µl) solution was added for 10 min incubation. At last, the optical density (OD) values at 490 nm were detected by a microplate reader (Molecular Devices Corp., Sunnyvale, CA, USA), which was regarded as the indicator for the analysis of cell proliferation. The MTT assay was repeated three times with similar results.

***AnnexinV-FITC/PI double staining***

Eca109 cells were digested with trypsin, centrifuged, and made into single cell suspension (1×10^6^ cells/ml) by using the buffer solution (calcium-containing PBS). Next, the cell suspension (100 μl) was transferred into a tube, followed by the addition of 5 μl AnnexinV-FITC and 5 μl propidium iodide (PI) for incubation at 4 °C for 30 min. Then, flow cytometry (Beckman-Coulter, CA, USA) was immediately used to detect cell apoptosis after the addition of a binding buffer (400 μl), followed by data analysis using Cell Quest software (Becton Dickinson, CA, USA). This experiment was repeated independently in triple.

***Wound-healing assay***

Eca109 cells were placed onto the 6-well plate for incubation at 37 °C. When cells covered the bottom of the plate, the scratch lines across the center of the well were drawn by using a sterilized P200 tip, which were photographed and marked as 0 hr under an inverted microscope (Olympus America, Melville, New York, NY, USA). After incubation for 24 hr at 37 °C, cell culture fluid was removed followed by rinsing three times in PBS. Next, serum-free medium was added onto the plate, which was photographed (24 hr). The distance was measured by the Image J software (Scion Corp., MD, USA) and the migration rate was calculated. The assay was repeated in triple to obtain the mean value.

***Transwell assay***

The upper and lower Transwell chambers (Costar, Boddenheim, Germany) were added with pre-warmed medium at 37 °C for 2 hr. Then, 60–80 μl matrigel was added into each chamber for incubation at 37 °C for 30 min until solidification of matrigel. Then, the complete medium (0.5 ml) was added onto the plate followed by the addition of cell suspension (0.5 ml, 5×l0^4 ^cells/ml) into the upper chamber for incubation at 37 °C for 24 hr. Then, the cells on the surface were removed, and the plate was rinsed with PBS. Next, after fixing with cold methanol for 30 min, the cells migrating to the lower Transwell chamber were stained with 0.1% crystal violet solution for 10 min, and the number of cells was observed under an Olympus inverted microscope (Melville, New York, NY, USA) by using six visual fields. The procedure of Transwell invasion assay was repeated three times.

***Statistical method***

The SPSS software (version 22.0, Chicago, IL, USA) was used to statistically analyze the data in this study. Measurement data were exhibited by mean ± standard deviation (mean±SD). The differences between the two groups were evaluated by using the Student’s *t*-test. The comparison among more than two independent groups was performed by one-way analysis of variance (ANOVA) with a Tukey’s *post hoc* test. *P*<0.05 indicated the significant difference.

## Results

***Propofol blocked the Wnt/β-catenin pathway in H***_2_ ***O***_2_ ***-induced Eca109 cells***

**Figure 1:**
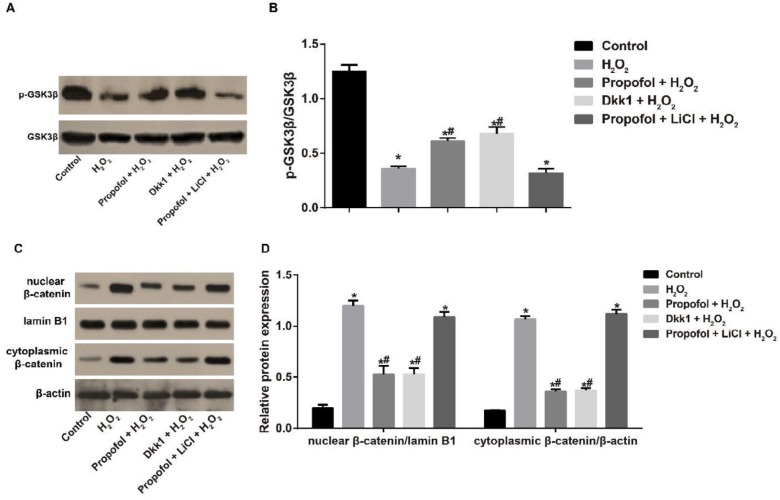
Effect of propofol on the Wnt/β-catenin signaling pathway in hydrogen peroxide-induced Eca109 cells

Note: A-B, The expression of GSK3β in Eca109 cells detected by Western blotting. C-D, Western blotting analysis determined the nuclear and cytoplasmic expression of β-catenin in Eca109 cells. * *P*<0.05 compared with the Control group; # *P*<0.05 compared with H_2_O_2_ and the Propofol + LiCl + H_2_O_2_ groups

According to the results of Western blotting demonstrated in Figure 1, H_2_O_2 _significantly up-regulated the nuclear and cytoplasmatic expressions of β-catenin, but down-regulated the p-GSK3β expression (all *P<*0.05). However, propofol could apparently restore β-catenin nuclear accumulation, but elevate p-GSK3β in H_2_O_2_-induced Eca109 cells, which was similar to Dkk1 (all *P<*0.05). Although the nuclear and cytoplasmatic expressions of β-catenin were lower and p-GSK3β was higher in the Dkk1 + H_2_O_2_ group (all *P<*0.05), there was no difference from the propofol + H_2_O_2_ group (all *P>*0.05). In addition, propofol can effectively inhibit the activation of the Wnt/β-catenin pathway caused by LiCl in the H_2_O_2_-induced Eca109 cells (*P<*0.05). Moreover, there was no statistical difference among any groups of cells regarding the expression of total β-catenin and total GSK3β (all *P>*0.05). 

**Figure 2:**
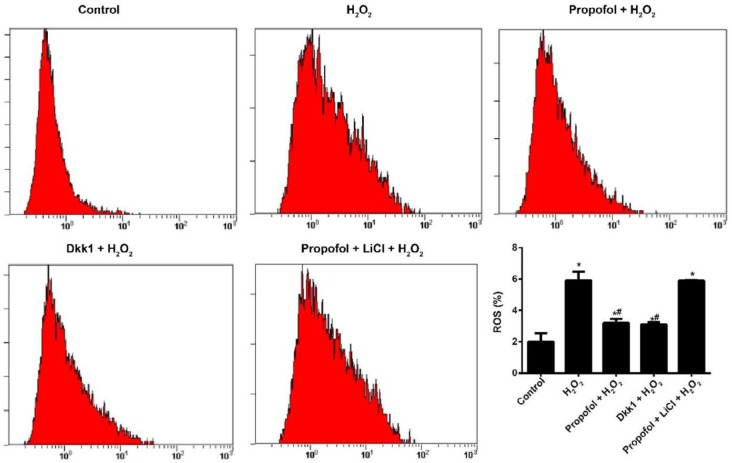
Effect of propofol-mediated Wnt/β-catenin pathway on reactive oxygen species (ROS) content in hydrogen peroxide-induced Eca109 cells

Note: * *P*<0.05 compared with the Control group; # *P*<0.05 compared with H_2_O_2_ and the Propofol + LiCl + H_2_O_2_ groups

**Figure 3:**
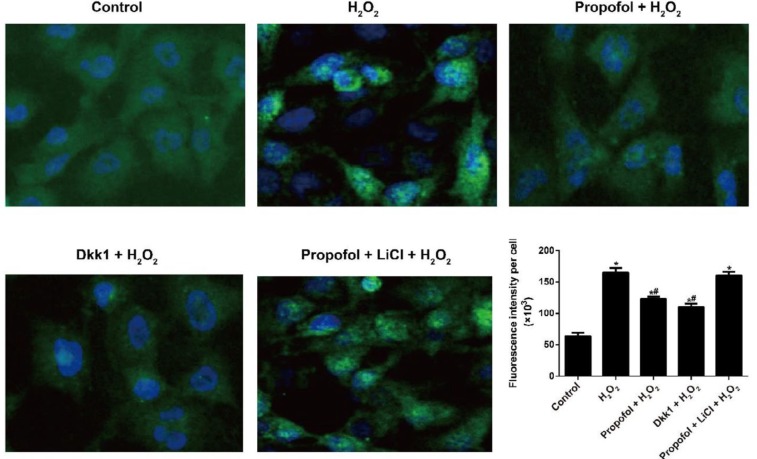
Effect of propofol-mediated Wnt/β-catenin pathway on the oxidative DNA damage of hydrogen peroxide-induced Eca109 cells

Note: * P<0.05 compared with the Control group; # *P*<0.05 compared with H_2_O_2_ and Propofol + LiCl + H_2_O_2_ groups

**Figure 4:**
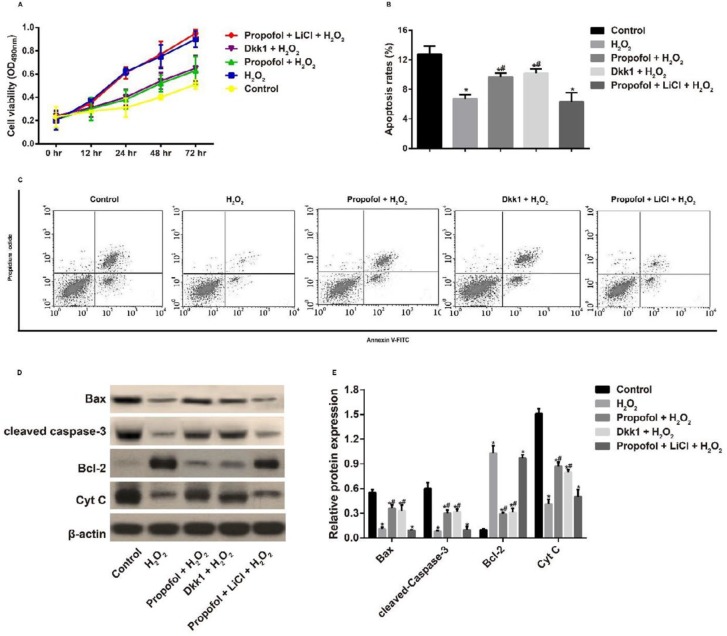
Effect of propofol-mediated Wnt/β-catenin pathway on the proliferation and apoptosis of hydrogen peroxide-induced Eca109 cells

Note: A, Cell proliferation in each group detected by MTT assay; B-C, Cell apoptosis in each group measured by AnnexinV-FITC/PI double staining; D-E, The expression of mitochondrial apoptosis-related proteins (including Cyt C, Bax, cleaved caspase-3, and Bcl-2) in each group determined by Western blotting; * *P*<0.05 compared with the Control group; # *P*<0.05 compared with H_2_O_2_ and the Propofol + LiCl + H_2_O_2_ groups

**Figure 5:**
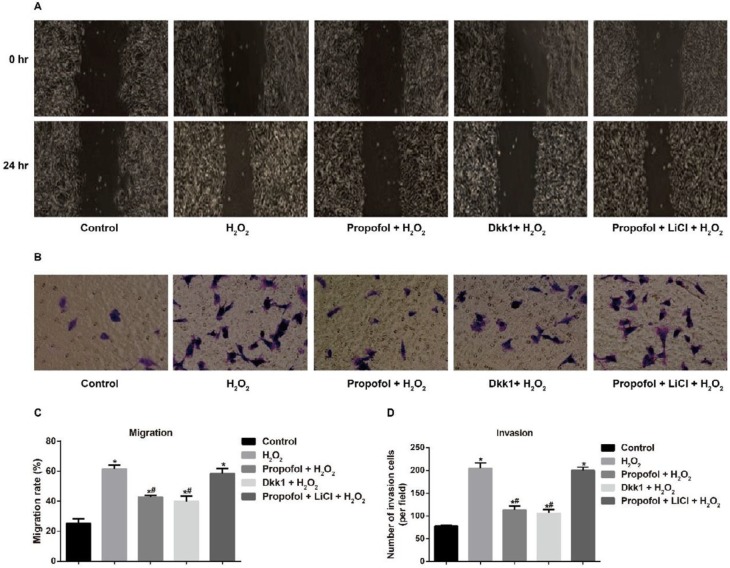
Effect of propofol mediating Wnt/β-catenin pathway on the migration and invasion of hydrogen peroxide-induced Eca109 cells

Note: A, C: Cell migration ability in each group evaluated by Wound-healing assay; B, D: Cell invasion ability in each group measured by Transwell assay; * *P*<0.05 compared with the Control group; # *P*<0.05 compared with H_2_O_2_ and the Propofol + LiCl + H_2_O_2_ groups

***Propofol alleviated oxidative stress and DNA damage in H***_2_ ***O***_2_ ***-induced Eca109 cells via inhibition of Wnt/β-catenin pathway***

H_2_O_2 _resulted in increase in the ROS content (all *P<*0.05), whereas propofol and Dkk1 could effectively inhibit the oxidative stress induced by H_2_O_2 _(*P<*0.05). Besides, in terms of the ROS content in Eca109 cells, the H_2_O_2_ group showed no significant difference with the propofol + LiCl + H_2_O_2_ group (all *P*>0.05, Figure 2). Moreover, H_2_O_2 _presented an obvious oxidative DNA damage in Eca109 cells when compared with the Control group (all *P<*0.05). However, propofol and Dkk1 remarkably alleviated the oxidative DNA damage in H_2_O_2_-induced Eca109 cells (all *P<*0.05). Furthermore, there was no statistical difference between the effects of propofol and Dkk1 on oxidative DNA damage in H_2_O_2 _- induced Eca109 cells (all *P>*0.05, Figure 3).

***Effect of propofol-mediated Wnt/β-catenin pathway on the proliferation and apoptosis of H***_2_ ***O***_2_ ***-induced Eca109 cells***

The H_2_O_2_ group had increased cell proliferation (24, 48, and 72 hr) and decreased cell apoptosis as compared with the Control group (all *P<*0.05). After treated with propofol or Dkk1, the proliferation was inhibited while the apoptosis was enhanced in H_2_O_2_-induced Eca109 cells (both *P<*0.05). Meanwhile, Eca109 cells in the propofol + LiCl + H_2_O_2_ group did not show any observable difference from cell proliferation and apoptosis in the H_2_O_2_ group (all *P>*0.05, Figures 4A-C). Additionally, we found that H_2_O_2_ could effectively suppress the expressions of cytochrome c (Cyt C), Bax, and cleaved caspase-3, but promote the Bcl-2 expression in Eca109 cells (all *P<*0.05), which were reversed by propofol or Dkk1 (all *P<*0.05). Moreover, H_2_O_2_ and propofol + LiCl + H_2_O_2 _groups had no observable differences concerning the expressions of mitochondrial apoptosis-related proteins (all *P*>0.05, Figures 4 D-E). 

***Propofol inhibited the migration and invasion of H***_2_ ***O***_2_ ***-induced Eca109 cells through mediating the Wnt/β-catenin pathway***

As shown in Figure 5, it is obvious that H_2_O_2 _could induce the migration and invasion of Eca109 cells in comparison with Controls (all *P<*0.05). Besides, both propofol and Dkk1 markedly reduced the metastasis of H_2_O_2_-induced Eca109 cells (all *P<*0.05), and Dkk1 slightly but not statistically decreased the migration and invasion of H_2_O_2_-induced Eca109 cells when compared with propofol (all *P>*0.05). In addition, Propofol + LiCl + H_2_O_2_ and H_2_O_2_ groups were not statistically different from each other regarding the cell abilities of migration and invasion (all *P>*0.05).

## Discussion

Cells may produce numerous ROS from essential metabolic processes to participate in regulating various physiological and pathological processes, like superoxide anion radical (O_2_-), hydroxyl radical (·OH), and H_2_O_2_ (26, 27). The increased oxidative stress could induce a broad spectrum of damage to biological systems, ultimately leading to different types of diseases, such as cardiovascular diseases, neurodegenerative diseases, and tumors (28). Therefore, better understanding the regulatory mechanism of anti-oxidative stress may contribute to the improvement of the therapeutic effect of ESCC. H_2_O_2_, an important relatively stable non-radical ROS, is usually used in the investigation of oxidative stress, since it can diffuse freely to the nucleus to induce DNA base damage and strand breakage (29, 30). As reported, ROS has pro-cancer activities, including promoting cell proliferation, angiogenesis, and metastases, as well as suppressing apoptosis (31). Researchers also found H_2_O_2 _could sequentially activate the ERK2 MAPK, NF-kappaB1 p50, and NOX5-S, and enhance the production of ROS, thus increasing the proliferation of EAC cells (32). Besides, the H_2_O_2 _-induced migration and invasion could be reversed using curcumin in pancreatic cancer cells by blocking the ROS/ERK/NF-kappaB signaling pathway (33). From our models, we found that H_2_O_2_ significantly increased the ROS content and caused oxidative DNA damage of Eca109 cells with elevated cell proliferation and metastasis, as well as decreased cell apoptosis, showing the successful construction of oxidative cell damage models in our research. 

Propofol has been widely used clinically for anesthesia and sedation due to its safe and excellent anesthetic effects (34). A large number of studies found the antioxidant properties of propofol. For example, Romuk *et al. *has confirmed the neuroprotective effects of propofol in the brain by controlling oxidative stress (35). A study by Romuk revealed that the antioxidative protection of propofol in PC12 cells was Ca^2+^-dependent and was mediated by NADPH oxidase (36). Worth mentioning, our study also revealed that propofol could remarkably alleviate the oxidative stress and DNA damage in Eca109 cells induced by H_2_O_2_. Accumulating evidence has pointed out the influence of propofol on the activity of tumor cells. As reported by researchers, propofol could inhibit the transcriptional activity of androgen receptors, as well as cell proliferation, in human prostate cancer cells (37). Ecimovic and his colleague also revealed that propofol could inhibit the breast cancer cell migration, but was reversed by down-regulation of NET1 (38). In our study, propofol could also dramatically suppress the proliferation and metastasis of H_2_O_2_-induced Eca109 cells, effectively promote cell apoptosis. Consistently, Ou *et al. *also demonstrated a similar trend of propofol in Eca109 cells (15). There was a possible explanation that propofol can reduce the production of ROS by the NADPH oxidase enzymatic system, thus act as a pro-apoptotic, anti-proliferative, and anti-metastasis stimulus in ESCC.

Notably, the Wnt/β-catenin pathway has attracted increasing attention from scholars in recent reports concerning the aspect of oxidative stress. Not surprisingly, H_2_O_2 _in our study could activate the Wnt/β-catenin pathway in Eca109 cells. As we know, oxidative stress generated by treatment of cells with H_2_O_2 _can activate the expressions of Foxo transcription factors (like Foxo1, Foxo3a, and Foxo4), which may interact with β-catenin to inhibit the apoptosis of cancer cells (39). Besides, the Wnt pathway is believed to be one of the major targets of p53 and p53-β-catenin pathways, playing an important role in the regulation of DNA damage and cell apoptosis (40, 41). In addition, it is well documented that the Bcl-2 family members (the arbiters of mitochondrial apoptotic pathway), derived from BH3 domains, which can process with the pro-apoptotic Bcl-2 members (like Bax and Bak) to release Cyt C (42, 43), thereby agglomerating procaspase-9 in cytoplasm and activating downstream Caspase-3, eventually inducing cell apoptosis (44, 45). Actually, our study also observed the promoted proliferation and inhibited apoptosis of Eca109 cells treated by H_2_O_2 _with the decreased expression of Cyt C, Bax, and cleaved caspase-3, and the increased expression of Bax. Moreover, consistent with the findings of a previous study (33), H_2_O_2_ could facilitate the abilities of migration and invasion of Eca109 cells in our study, which indicated the possibility that H_2_O_2_ could activate the Wnt/β-catenin signaling pathway and promote the epithelial-mesenchymal transition (EMT) (46), thereby promoting metastasis of EC cells. Moreover, propofol was found to block the Wnt/β-catenin signaling pathway in H_2_O_2_-induced Eca109 cells, which was consistent with the study reported previously (15). Coincidently, another study demonstrated that propofol could inhibit the Wnt/β-catenin pathway and reduce β-catenin expression in metastatic tumor tissues, eventually blocking the pulmonary metastasis of MADB106 tumor cells in tumor-bearing rats (47). Last but not least, propofol in our study was also shown to inhibit the effect of LiCl on activating the Wnt/β-catenin signaling pathway in H_2_O_2_-induced Eca109 cells, which suggested that propofol may play a protective role in H_2_O_2_-induced ESCC cells by blocking Wnt/β-catenin signaling pathway.

## Conclusion

Collectively, our study may suggest that propofol exerts a protective role in H_2_O_2_-induced Eca109 cells possibly by blocking the Wnt/β-catenin signaling pathway.
